# Impact of on-site mobile CT imaging on prehospital patient management during the Munich Oktoberfest: a retrospective observational before–after study at a mass gathering

**DOI:** 10.1186/s12873-026-01785-4

**Published:** 2026-10-06

**Authors:** Wilhelm Flatz, Dominik Hinzmann, Philip Kampmann, Leonhard Poehlmann, Paul Reidler, Jenny Schlichtiger, Karl-Georg Kanz, Jeffrey Bazarian, Jens Ricke, Viktoria Bogner-Flatz

**Affiliations:** 1 Department of Radiology, LMU University Hospital, LMU Munich, Munich, Germany; 2 Department of Clinical Medicine, Department of Anesthesiology and Intensive Care Munich, TUM School of Medicine and Health, Munich, Germany; 3 Emergency Medical Service Authority, City of Munich and greater Munich area, Munich, Germany; 4 Aicher Ambulance Emergency Medical Service Munich, Munich, Germany; 5 Department of Trauma and Reconstructive Surgery, MUM, LMU University Hospital, LMU Munich, Munich, Germany; 6 Department of Internal Medicine I – Cardiology, University Hospital, LMU Munich, Munich, Germany; 7 EMS Authority Upper Bavaria, Munich, Germany; 8 Department of Emergency Medicine, University of Rochester School of Medicine and Dentistry, Rochester, NY USA; 9 Department of Trauma Surgery, TUM School of Medicine and Health, Munich, Germany; 10 Ärztliche Bezirksbeauftragte Rettungsdienst, Rettungsdienstbezirk Oberbayern West, Regierung von Oberbayern, Sachgebiet 10 – Sicherheit und Ordnung, Geschäftsstelle Ärztlicher Leiter Rettungsdienst, Maximilianstr. 39, 80538 München, Germany

**Keywords:** Mobile computed tomography, Mobile CT, Imaging, Oktoberfest, Mass gathering, mTBI, TBI, Munich, Emergency medical services, EMS

## Abstract

**Background:**

Mass gatherings such as the Munich Oktoberfest increase demand on Emergency Medical Services (EMS), particularly for trauma patients requiring imaging. Suspected mild traumatic brain injury (mTBI) often necessitates hospital referral and diagnostic imaging, placing strain on prehospital and hospital resources.

**Methods:**

This retrospective observational before–after study evaluated the impact of an on-site mobile computed tomography (CT) scanner deployed during the Oktoberfest in 2022 and 2023. Patients with suspected head, facial, or cervical spine trauma requiring CT imaging were included. EMS hospital transport data during these events (with mobile CT) were compared with a historical reference period from 2015 to 2019 (without mobile CT) and with matched pre- and post-event periods. Standardized prehospital protocols guided patient assessment and imaging decisions.

**Results:**

A total of 498 patients underwent on-site CT imaging. Of these, 429 (86%) were treated on-site and discharged after imaging and clinical evaluation, while 52 (10%) required hospital transport based on CT findings. Additional transports occurred for unrelated medical issues. On-site CT availability prevented 429 EMS transports, corresponding to an estimated 22,737 min of EMS deployment time. Trauma-related hospital admissions during Oktoberfest were lower in 2022–2023 than in 2015–2019, and the relative increase in admissions during the event was reduced.

**Conclusion:**

This retrospective analysis of the use of a mobile CT scanner in a prehospital setting during a mass gathering found that its implementation was associated with improved patient triage, fewer hospital transports, and more efficient EMS resource utilization. While the retrospective before–after study design does not permit direct causal inference, on-site imaging may have contributed to more targeted patient management and reduced demand on hospital resources. These findings suggest that mobile CT could represent a useful model for supporting medical services at large-scale public events and warrant further prospective evaluation.

**Supplementary Information:**

The online version contains supplementary material available at https://doi.org/10.1186/s12873-026-01785-4.

## Introduction

The Munich Oktoberfest, the largest fair in the world [1, 2], attracts millions of visitors to the city each year over a period of approximately two weeks, resulting in a high concentration of attendees in a confined urban area. First held in 1810, the festival has only been canceled under exceptional circumstances, such as the World Wars and the COVID-19 pandemic in 2020 and 2021. The pandemic has posed substantial challenges to Emergency Medical Services (EMS) and healthcare systems worldwide [3], contributing to ongoing limitations in hospital capacity.

Attendance at large-scale events such as Oktoberfest is associated with a wide range of medical risks [4–7] and presents recurring challenges for prehospital emergency care [8]. To address these demands, an extended on-site medical service staffed by paramedics and emergency physicians is deployed at the festival site. The most common reasons for seeking medical care include alcohol intoxication, trauma—particularly head and maxillofacial injuries—as well as cardiovascular and neurological symptoms such as impaired consciousness, dizziness, and disorientation [9].

Head and facial injuries represent a particular diagnostic and logistical challenge in this setting, as analyzed in over 500.000 emergency cases in Munich [10]. Potential complications such as intracranial hemorrhage or facial fractures require computed tomography (CT), which remains the diagnostic gold standard [11]. Management of these patients is frequently complicated by intoxication, language barriers, and limited compliance, increasing the demand on both medical personnel and emergency services [4, 12]. At the same time, transport to hospital for imaging contributes to the burden on prehospital and in-hospital resources.

In response to these challenges, Munich EMS implemented an enhanced emergency response concept during Oktoberfest that included the use of an on-site mobile CT scanner. The aim of this approach was to enable advanced imaging directly at the event site, improve patient care, and reduce the need for hospital transport while maintaining hospital-equivalent diagnostic standards [13].

The aim of this retrospective observational before–after study was to evaluate the impact of an on-site mobile CT scanner on the management of patients with suspected mild traumatic brain injury requiring head, facial, or spinal imaging during Oktoberfest. It was hypothesized that the availability of mobile CT would enhance prehospital diagnostic capacity, optimize patient flow, and reduce the need for hospital transport.

The conception, implementation, and operational aspects of the Munich Oktoberfest mobile CT program, together with the initial experience from its first deployment in 2022, have previously been reported by our group [13, 14]. However, those publications focused on proof-of-concept and implementation rather than a comprehensive clinical evaluation. The present study expands this work by including patients from both the 2022 and 2023 Oktoberfest periods and by providing detailed analyses of patient characteristics, imaging findings, EMS utilization, transport decisions, and clinical outcomes.

## Methods

### Ethical approval

This study was conducted in accordance with the Declaration of Helsinki. Ethical approval for this retrospective analysis of anonymized data was granted by the Institutional Review Board of Ludwig Maximilian University (approval numbers 20–258 and 22–0885). The requirement for informed consent was waived due to the retrospective design and use of anonymized data.

### Munich’s Oktoberfest and the IEHS

During the 16 days of Munich’s Oktoberfest 2023 – held, as always, at the “Theresienwiese” in central Munich, covering a total area of 42 hectares - visitors had access to 38 hectares of festival grounds. These grounds accommodated 503 businesses, including food vendors, amusement operators and market traders.

Seventeen large temporary festival tents - each measuring up to 100 × 50 m and equipped with stages, beer taps, kitchens, and second-floor balconies - can host up to 12,000 visitors at once. In addition, 21 smaller tents accommodate up to 2,000 visitors each [15]. Beer consumption during Oktoberfest is estimated at 5 to 8 million liters annually.

On-site medical services (IEHS) are centrally coordinated and provided at the medical service center (“Sanitätswache”), located in the “Behördenhof” - a large, secured complex that also houses fire, police, and other emergency services. Positioned on the western edge of the Theresienwiese, the Behördenhof facilitates rapid deployment to any location within the festival grounds.

In 2022 and 2023, a CT scanner was installed and operated at this location. Thousands of patients are treated here annually for conditions such as alcohol intoxication, lacerations, mild traumatic brain injury (mTBI) and various other medical issues [16].

IEHS operations commence three hours before the official opening on the first day and run continuously 24/7 until the end of the 16- to 18-day event. This includes late-night interventions and continuous monitoring, particularly of intoxicated individuals.

Functionally, the IEHS resembles a small emergency department, albeit with limited diagnostic resources. However, in addition to clinical examinations, blood gas analysis (BGA), ultrasound, and since 2022 computed tomography (CT) are available. Minor injuries are often treated on-site, avoiding unnecessary hospital transfers.

To ensure comprehensive coverage, up to 140 healthcare professionals are on duty daily, ranging from emergency medical technicians (EMTs) with Basic Life Support (BLS) certification to paramedics and experienced emergency physicians. For advanced care and transport, up to 15 ambulances (RTWs), 10 patient transport vehicles (KTWs), and 3 emergency physician response vehicles (NEFs) are available, covering both the festival and surrounding areas.

### Mobile CT imaging

In 2022 and 2023, a mobile CT scanner (Siemens Healthineers, Erlangen, Germany) was installed in a shielded container adjacent to the on-site medical service center. Prior to deployment, all required governmental assessments and technical approvals were obtained. The system was operated during peak hours and staffed by trained radiology personnel.

**Fig. 1:**
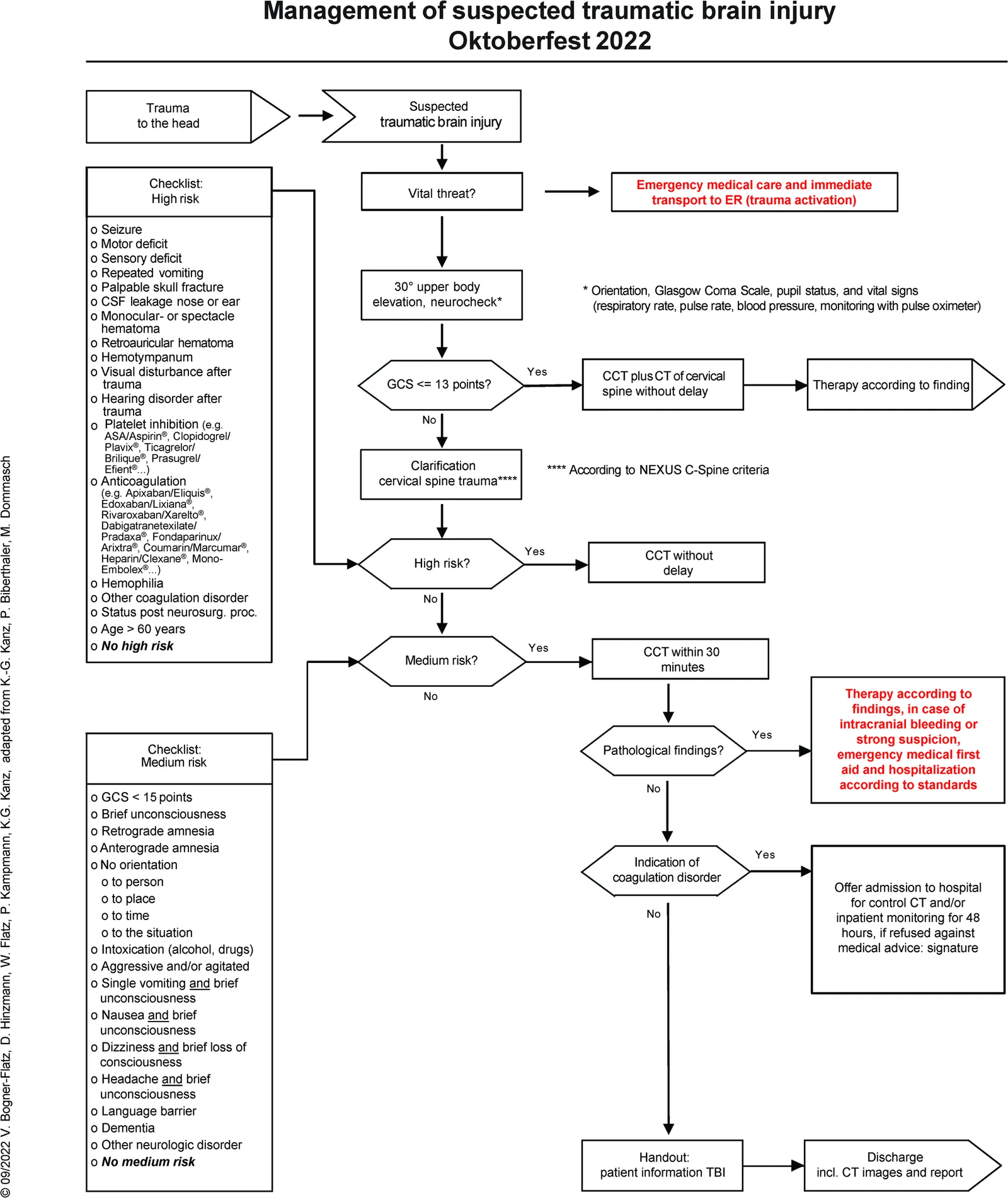
Algorithm for clinical decision making

Patients with suspected head, facial, or cervical spine trauma underwent CT imaging based on standardized clinical decision rules adapted from the NEXUS criteria and the Canadian CT Head Rule. Non-contrast CT scans of the head, maxillofacial region, and/or cervical spine were performed. Baseline characteristics (vital signs, injury mechanism, associated injuries, Glasgow Coma Scale [GCS], signs of intoxication) were documented. Patients with moderate to severe trauma or polytrauma were excluded from on-site imaging and referred directly to hospital care. The treatment algorithm for these patients is shown in Fig. 1. This figure illustrates the clinical algorithm that has been developed for CT decision making at Oktoberfest medical center on the festival grounds. It is essentially composed from a well established clinical algorithm (34) as a combination of NEXUS Head CT Instrument and Canadian CT Head Rule.

All scans were evaluated by senior radiologists. Based on imaging and clinical findings, patients were either treated on-site or referred for hospital admission. The city’s rescue coordination center tracked patient outcomes, including on-site discharge or hospital transfer. Clinical outcomes were classified as discharge, hospital admission (conservative or surgical), complete recovery or recovery with limitations. Clinical outcomes were assessed using available discharge records.

### EMS admission data

Hospital transport data were obtained from the Interdisciplinary Medical Care Capacity Management System (IVENA), which records EMS patient transfers and hospital admissions in Munich. Data were collected for the Oktoberfest periods in 2022 and 2023, as well as for matched pre- and post-event periods within the same years.

To allow comparison, historical data from 2015 to 2019 (without mobile CT availability) were included as a reference group. Data were normalized to a standardized observation period to account for variations in festival duration. Data were adjusted to reflect mobile CT scanner operating hours. Figure 6 presents an overview of the hospital infrastructure in Munich.

### Statistical analysis

Statistical analyses were performed using SPSS Statistics (Version 29, IBM Corp.) and Microsoft Excel 2016. Descriptive statistics were calculated, and normality was assessed using the Shapiro–Wilk test. Depending on data distribution, group comparisons were performed using the Mann–Whitney U test or the Kruskal–Wallis test. A p-value ≤ 0.05 was considered statistically significant.

## Results

### Study population

Attendance at Munich’s Oktoberfest increased from 5.7 million visitors in 2022 to 7.2 million in 2023. The Event-to-Host Population Ratio (EHP) was 3.86 in 2022 and 4.76 in 2023 [17], while for 2015–2019, the corresponding EHP values were 4.07, 3.78, 4.16, 4.14, 4.28 (mean = 4.09, SD = 0.18, range 3.78–4.28). In 2020 and 2021 Oktoberfest was canceled due to the pandemic. A total of 5,444 patients were treated on-site in 2022 and 8,157 in 2023 [18, 19], corresponding to a Patient Presentation Rate (PPR) per 10,000 visitors of 9.6 and 11.3 respectively, for 2015–2019, PPR values were 12.4, 12.1, 12.9, 12.7 and 12.5 (mean = 12.52, SD = 2.9, range 12.1–12.9).

Overall, 498 patients with mild traumatic brain injury (mTBI) underwent on-site CT imaging, including 413 (83%) male patients. Cranial CT (CTH) only was performed in 238 patients (48%), combined cranial and maxillofacial CT (MFCT) in 124 (25%), cranial and cervical spine CT (CSCT) in 76 (15%), and combined CTH, MFCT, and CSCT in 41 (8%). MFCT only was performed in 15 patients (3%) and CSCT only in 4 (1%). In total, 767 CT scans were acquired, including 470 cranial (61%), 178 maxillofacial (23%), and 119 cervical spine CTs (16%).

**Fig. 2:**
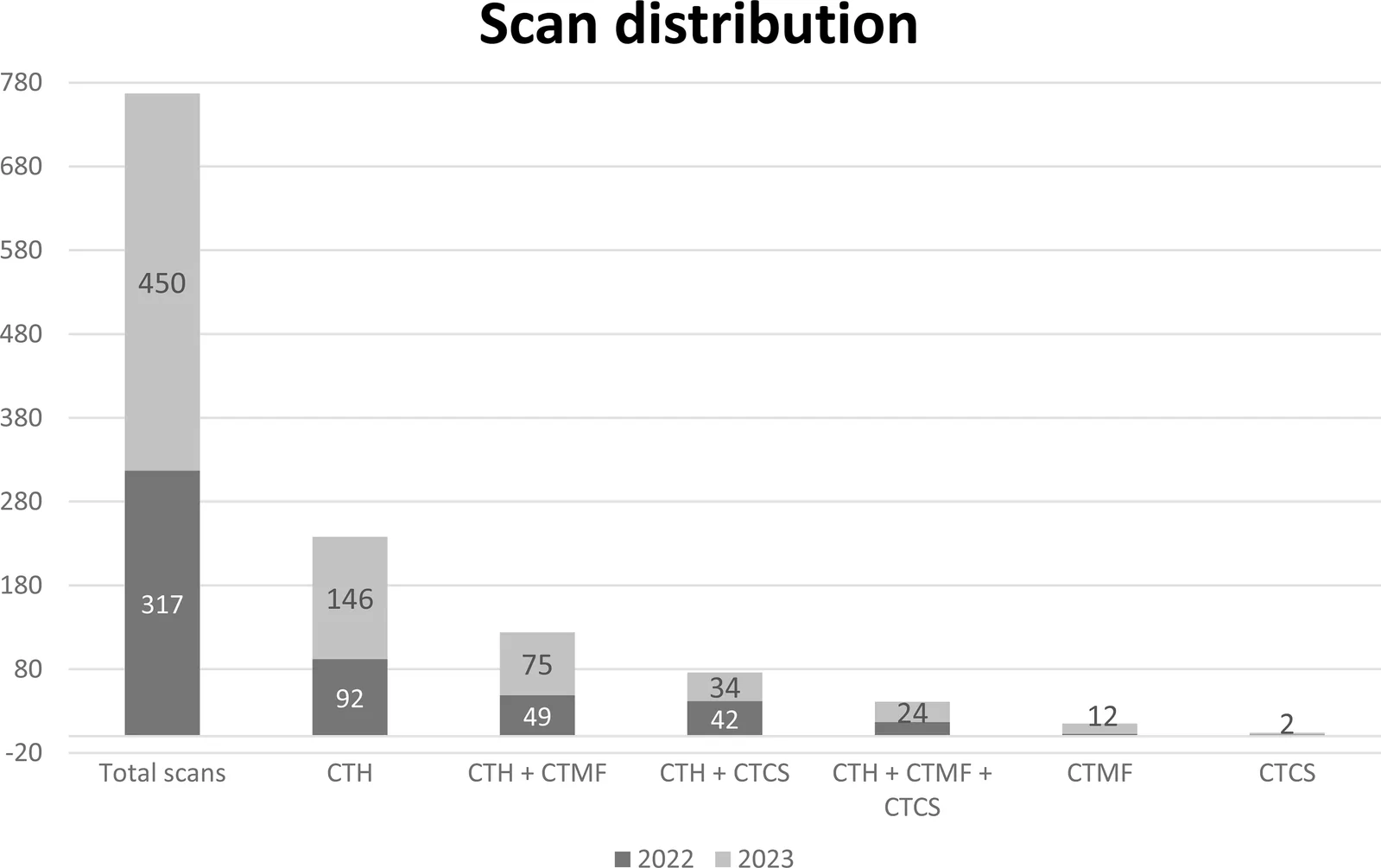
CT scan distribution by patients. Number of patients examined in the on-site CT scanner in 2022 and 2023

Intracranial bleeding was identified in 25 patients (5%) (2022: 11; 2023: 14), and midface fractures in 63 (13%) (2022: 23; 2023: 40), with combined findings in 4 patients (1%). No cervical spine injuries were detected. Glasgow Coma Scale (GCS) scores showed no relevant differences between groups (see Table 1; Fig. 2).

**Table 1:** Clinical baseline characteristics

	2022	2023	Total
Patients in CT scanner	205	293	498
**Gender**			
Male	174	241	415
Female	31	52	83
**Total scans by scanned area**	317	450	767
CTH	191	279	470
CTMF	67	111	178
CTCS	59	60	119
**CT Scans by patient**,** only**			
CTH	92	146	238
CTH + CTMF	49	75	124
CTH + CTCS	42	34	76
CTH + CTMF + CTCS	17	24	41
CTMF	3	12	15
CTCS	2	2	4
**cranial bleeding**	11	14	25
**gender of bleedings**			
Male	10	12	22
Female	1	2	3
**Midface fracture**	23	40	63
Transport due to CT	25	27	52
**Transport (total)**	31	38	69
**Clinical course of the transports**			
Outpatient	2	4	6
Inpatient	23	25	48
Discharge against medical advice	6	9	15
**Main transport reasons**			
TBI	11	14	25
Midface fracture	14	11	25
Intoxication	6	10	16
Others	0	3	3
**Saved transfers**	174	255	429
Saved transfer in minutes	9222	13,515	22,737

Based on CT findings, 52 patients (10%) required hospital admission and 17 (3%) were admitted for non-trauma-related reasons. Overall, 69 patients (14%) were transported to hospital, while 429 (86%) were managed on-site. Reasons for transport included intracranial bleeding in 25 cases (36%), craniofacial fractures in 25 (36%), severe intoxication in 16 (23%), and other causes in 3 (4%). Among transported patients, 48 (69%) required inpatient treatment, 15 (22%) were discharged against medical advice, and 6 (9%) received outpatient care.

### Comparative analysis and EMS utilization

**Fig. 3:**
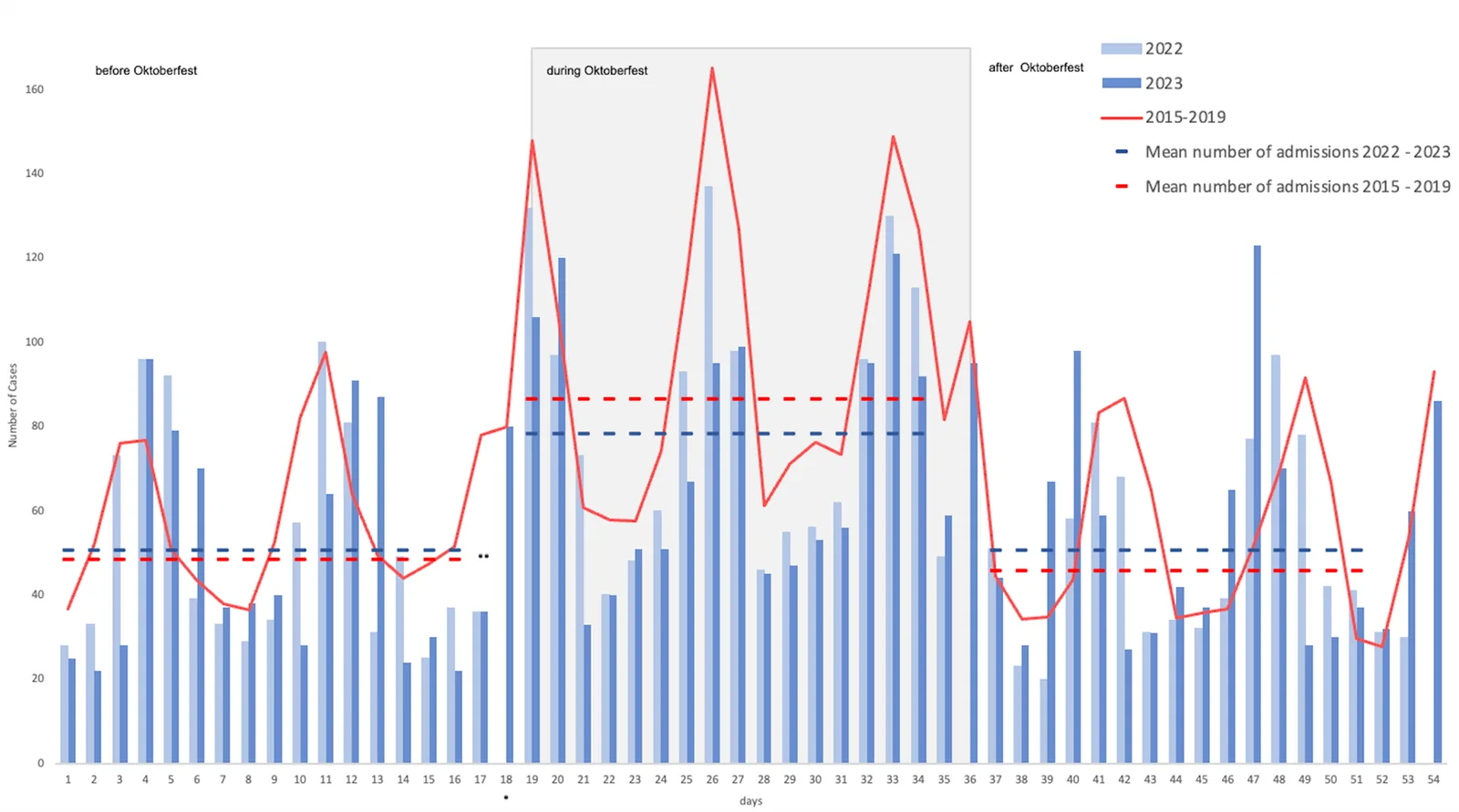
This graph shows the comparison of surgical emergency hospital admissions immediately before, during, and immediately after Oktoberfest in the years 2015–2019 vs. 2022–2023. In 2015–2019, admissions during Oktoberfest exceeded the time before and after (red dotted line). In 2022–2023, overall surgical admissions are lower than before and after OF – due to the CT on scene (blue dotted line). All data are adjusted to the CT operating hours. *data only available for 2016 (17 days),* 2017 (18 days)*, 2022 (17 days) ** for comparability of the data only 16 days were included

Trauma-related EMS hospital admissions during Oktoberfest were compared between 2015 and 2019 and 2022–2023. Admissions increased by 46% during Oktoberfest in 2015–2019 compared to pre- and post-event periods, versus 35% in 2022–2023 (2022: 39%; 2023: 32%) (see also Fig. 3).

**Fig. 4:**
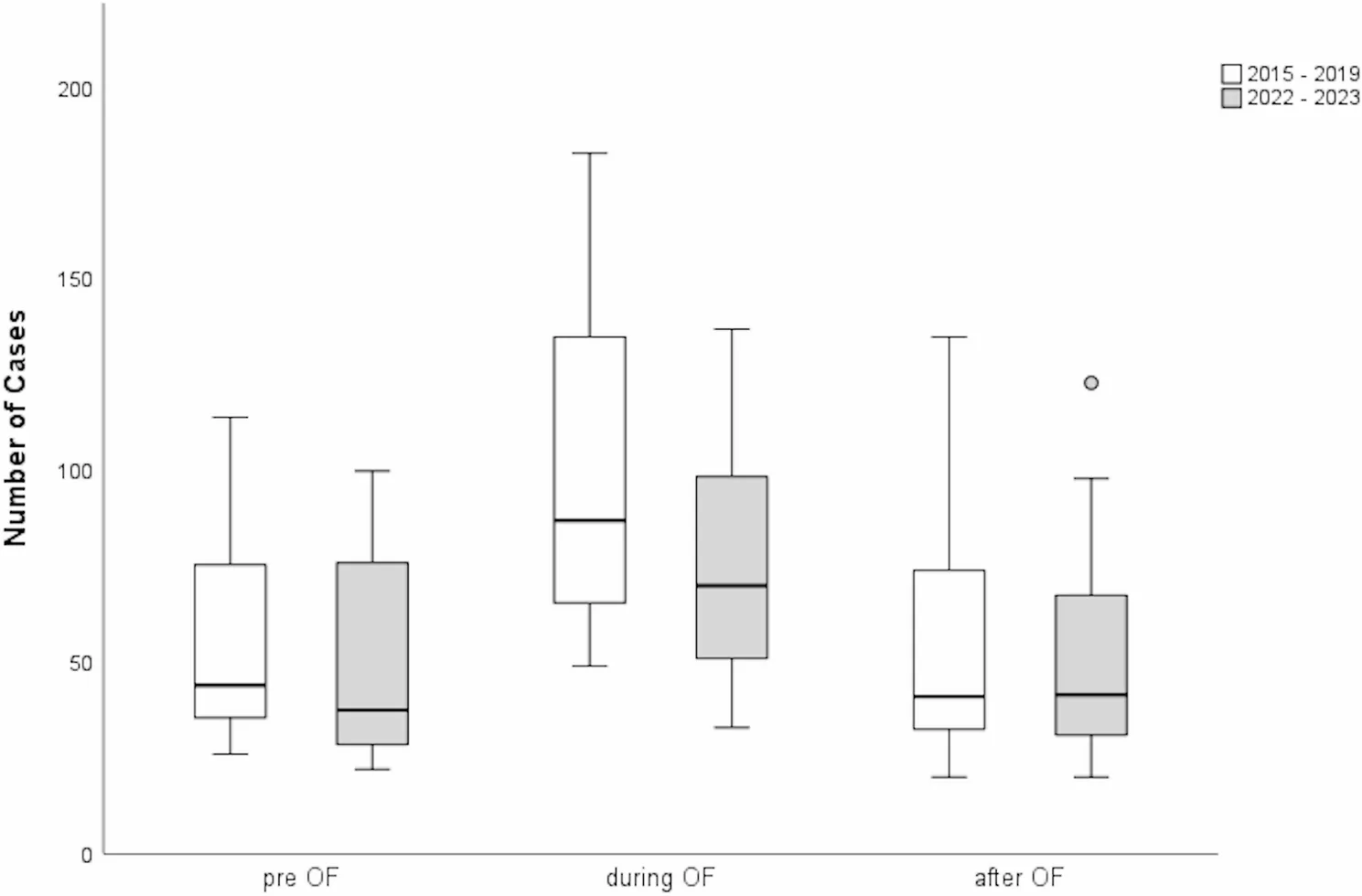
Comparison of trauma hospital admissions pre-, during-, and post Oktoberfest stratified by study period (2022–2023 vs. 2015–2019) Boxplots: box = interquartile range; middle line = median, whiskers = minimum/maximum, circles = outliers

Median hospital admissions were higher in 2015–2019 than in 2022–2023 (63.5 [IQR 38.5–90.0] vs. 51.0 [33.0–85.5]; *p* = 0.037). Median daily admissions during Oktoberfest were 87 (65.3–135) in 2015–2019 and 70 (51.0–98.8) in 2022–2023 (*p* = 0.004). In both periods, admissions increased significantly during Oktoberfest compared to pre- and post-event phases (all *p* ≤ 0.001), see Fig. 4.

**Fig. 5:**
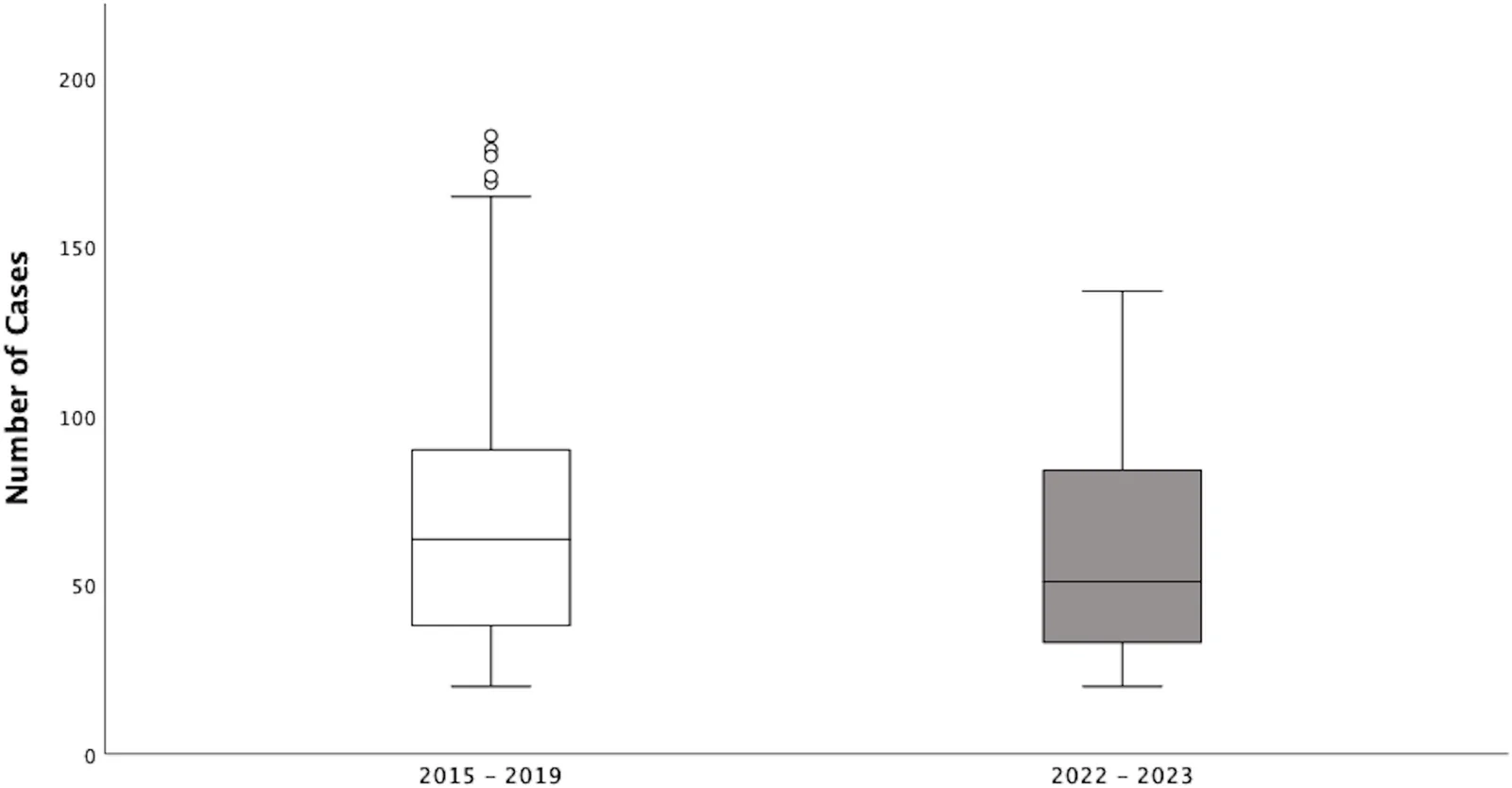
Overall comparison of trauma hospital admissions 2022–2023 vs. 2015–2019 (considering the overall study period pre-, during-, and post Oktoberfest) Boxplots: box = interquartile range; middle line = median; whiskers = minimum/maximum; circles = outliers

**Fig. 6:**
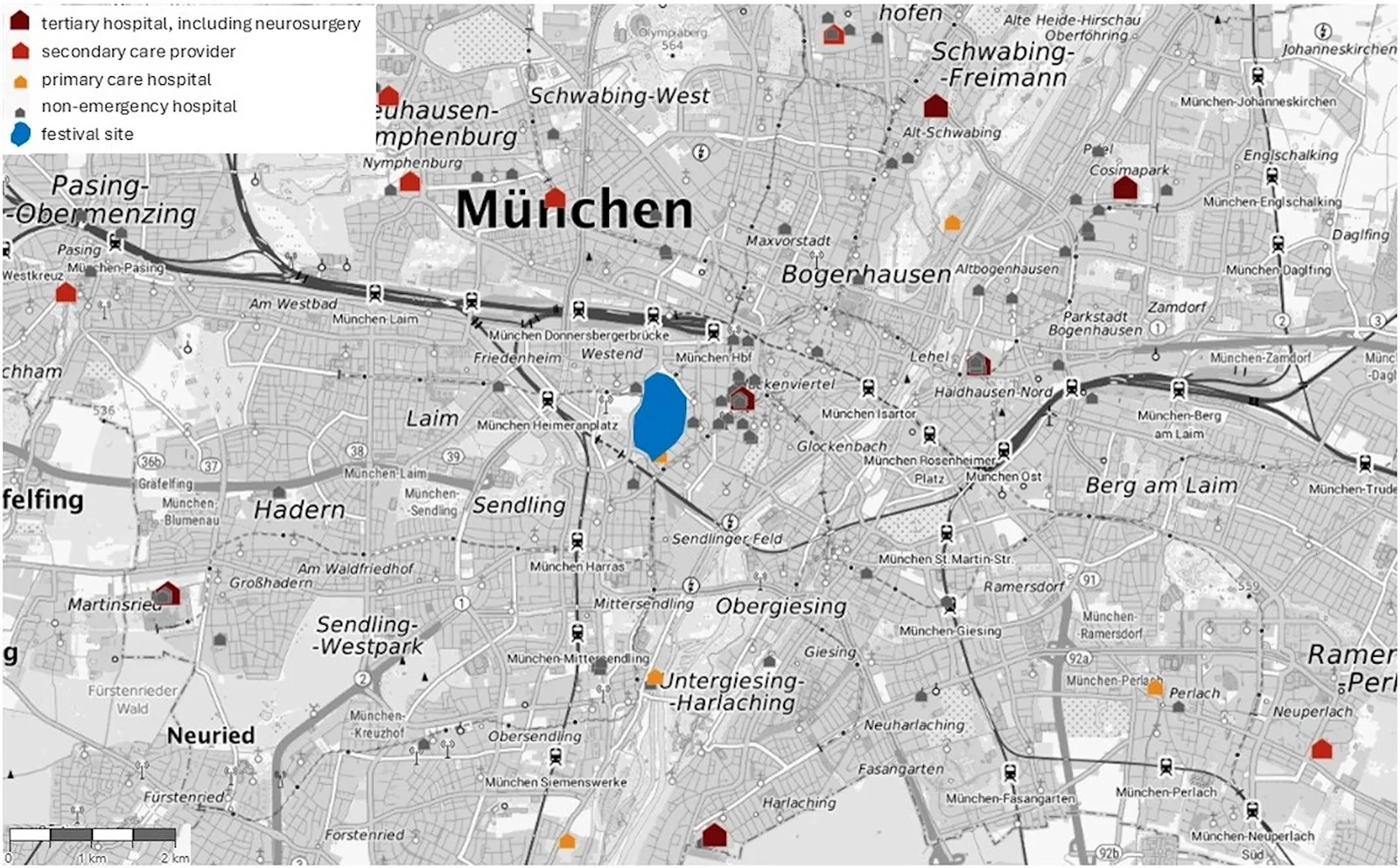
Overview map of the hospital infrastructure in Munich, illustrating the listed hospitals according to their care structure and distance to the fairground. Retrieved in April 2026 from https://datendienste.aelrd-bayern.de/ based on Federal Agency for Cartography and Geodesy (2026)

During 2022–2023, 429 patients (86%) were managed on-site without hospital transport. Based on an average EMS deployment duration of 53–54 min [20, 21], this corresponded to 9,222 min saved in 2022 and 13,515 min in 2023, totaling 22,737 min, equivalent to approximately 45 EMS shifts, see Fig. 5 and the layout of the hospital and the resulting travel times in Fig. 6. The total duration of 53–54 min corresponds to the average duration per year of all emergency medical service missions in the region. This value was also used for the Oktoberfest period; however, actual mission times may vary and are likely to be even longer in reality during the festival, when significantly more visitors are present in the city.

## Discussion

This study evaluates the implementation of an on-site mobile CT scanner during a large-scale mass gathering and its association with emergency medical service (EMS) utilization and hospital admissions. Among 498 patients with suspected mild traumatic brain injury (mTBI), the majority (86%) were managed on-site without hospital transport, while only 14% required transfer, primarily due to intracranial bleeding or craniofacial fractures. No cervical spine injuries were identified. These findings support the feasibility of on-site CT imaging as an effective triage tool in this setting.

Mobile CT has previously been used mainly to accelerate diagnosis in acute conditions such as stroke [22, 23] or to provide imaging in resource-limited environments [24]. Its application at mass gatherings has not been systematically studied. To our knowledge, this is the first study to assess its role in reducing strain on EMS and emergency departments (EDs) during a large recurring event.

The need for such strategies has increased following the COVID-19 pandemic, which has led to persistent healthcare system constraints in both Europe and the United States [25]. Overcrowding in emergency departments has become a growing global issue, contributing to increased patient risk and mortality [25]. In Munich, staffing shortages have reduced ambulance availability and hospital capacity, while only a limited number of centers can provide comprehensive traumatic brain injury diagnostics. During high-attendance events such as Oktoberfest, this creates a risk of system overload.

Compared with the pre-pandemic period (2015–2019), trauma-related hospital admissions during Oktoberfest were lower in 2022–2023, both in relative increase and absolute median values. Although admissions rose significantly during the event in all years, the magnitude of this increase was smaller in the period with on-site CT availability. However, the comparison between the two periods should be interpreted in the context of possibly broader organizational and societal changes occurring after the COVID-19 pandemic, including potential differences in healthcare utilization, emergency care pathways and visitor characteristics, which may also have influenced admission patterns. Nevertheless, the observed reduction in EMS utilization remained substantial during the study period. These findings are consistent with the possibility that immediate imaging may support more selective hospital referral.

The operational impact on EMS resources was substantial. A total of 429 patients were managed on-site without transport, corresponding to approximately 22,737 min of EMS deployment time saved. Given average mission durations of 53–54 min, this equals roughly 45 ambulance shifts. The duration of 53–54 min per deployment corresponds to the average overall mission duration of the emergency medical services in the city and district of Munich during the year. Although actual mission durations may vary, the substantial increase in population during Oktoberfest suggests that the selected average value represents the lower end of the typical range. These findings indicate that on-site CT can meaningfully reduce transport demand and help preserve limited prehospital resources during peak periods.

From a clinical perspective, intracranial bleeding was identified in 5% and midface fractures in 13% of patients, while most showed no findings requiring hospital-based care. A large proportion of patients were intoxicated, limiting the reliability of clinical examination and complicating triage decisions. In this context, CT imaging plays a critical role in safely excluding serious pathology [26, 27]. Many of these patients can be discharged after clinical assessment, provided that life-threatening conditions like intracranial bleeding are excluded [26]. Most patients managed on-site had no imaging findings indicating the need for immediate hospital-based treatment and were therefore discharged after clinical assessment and observation. Nevertheless, the absence of follow-up data precludes conclusions regarding delayed complications or longer-term clinical outcomes. Without on-site imaging, all patients would have required hospital transport for further evaluation. Notably, among those transferred, 69% required inpatient treatment, supporting the appropriateness of triage decisions.

While the primary aim of implementing mobile CT was to maintain system functionality rather than reduce costs, the observed reduction in transports and admissions suggests a relevant impact on resource utilization. Formal economic evaluation remains challenging within the German healthcare system due to separate funding structures for EMS and hospital care, although previous studies suggest that mobile CT may be cost-effective [28, 29].

Compared with other mass gatherings, the patient presentation rate observed in this study (0.96–1.13) was relatively low [30]. Existing literature on imaging in such settings is limited and focuses mainly on ultrasound or conventional radiography, typically for musculoskeletal injuries [31–33]. Data on CT use are lacking. While the baseline feasibility and operational framework of this on-site imaging program were established in our prior report [14], the data from the 2022–2023 cohorts presented here demonstrate its measurable clinical impact on patient management. The present study therefore adds novel evidence on the integration of advanced imaging into mass gathering medicine and its potential to reduce strain on emergency care systems.

### Limitations

This study has several limitations. Its retrospective and non-randomized design limits the ability to infer causality between on-site CT implementation and observed reductions in hospital admissions and EMS workload. The comparison of two years with mobile CT to historical data may introduce bias due to temporal changes in visitor behavior, alcohol consumption, or healthcare utilization patterns. Several key variables, including visitor numbers and intoxication levels, are based on estimates, which may limit the precision of associations. Additionally, organizational changes in on-site medical care, such as the introduction of overnight shifts in 2022–2023, may have influenced patient management. Finally, the study was conducted at a single site, which may limit generalizability to other mass gatherings or healthcare systems. Follow-up data for patients managed on-site were not available, restricting assessment of delayed complications or diagnostic safety.

## Conclusion

The integration of a mobile CT scanner during a large-scale mass gathering was associated with improved prehospital patient management-particularly those with suspected mild traumatic brain injuries or facial trauma, reduced hospital transports, and more efficient use of EMS resources. This approach may represent a valuable strategy for optimizing emergency care and alleviating pressure on healthcare systems during high-demand events.

## Supplementary Information

Below is the link to the electronic supplementary material.

Supplementary Material 1

Supplementary Material 2

## Data Availability

The datasets generated and/or analyzed during the current study are available from the corresponding authors on reasonable request.

## Abbreviations

mTBI: Mild traumatic brain injury
TBI: Traumatic brain injury
CT: Computed tomography
EMS: Emergency Medical Service
ED: Emergency department
OF: Oktoberfest
CTH: CT scan of the head
MFCT: Maxillofacial CT scan
CSCT: CT scan of the cervical spine
IVENA: Interdisciplinary Medical Care Capacity Management System
IEHS: In-Event Healthcare System
EHP: Event-to-host-population ratio
PPR: Patient Presentation Rate
EMT: Emergency medical technician
BLS: Basic Life Support
RTW: Ambulance (German abbreviation for Rettungswagen)
KTW: Patient transport vehicle (German abbreviation for Krankentransportwagen)
NEF: Emergency physician response vehicles (German abbreviation for Notarzteinsatzfahrzeug)
GCS: Glasgow Coma Scale
