# Supplementary material for: Impact of on-site mobile CT imaging on prehospital patient management during the Munich Oktoberfest: a retrospective observational before–after study at a mass gathering
Source: BMC Emerg Med. 2026 Oct 6;26:262. doi: 10.1186/s12873-026-01785-4 (PMC13644297; doi:10.1186/s12873-026-01785-4)
Supplement: Supplementary file 1 [file 12873_2026_1785_MOESM1_ESM.docx]

**Supplement 1:**

*CT Scan Protocol*

All scans were acquired 2022 using a commercially available container based CT scanner (SOMATOM go.TOP; Siemens Heathineers), no contrast agent was used.

For cranial CT scans (CTH) following parameters were used 120 kV, 286 ref. mAs, exposure modulation applied XCARE_ZEC, pitch 0.55, CTDIvol 37.18 mGy (sd. 2.65), mean DLP 631.38 mGy*cm (sd. 64.59), mean scan duration 8.45 s (sd. 0.47).

In 3 patients a fast CTH-scan protocol was used by changing pitch from standard 0.55 to 0.6.

For maxillofacial CT (MFCT) parameters were 70 kV, 78 ref. mAs, exposure modulation applied XCARE_ZEC, pitch 0.55, mean CTDIvol 7.80 mGy (sd. 3.61), mean DLP 98.00 mGy*cm (sd. 76.43), mean scan duration 6.66 s (sd. 0.85).

For cervical spine CT (CSCT) acquisition parameters were 140 kV, 208 ref. mAs, exposure modulation applied ELLIP_ZEC, pitch 0.8, mean CTDIvol 15.44 mGy (sd. 0.46), mean DLP 323.24 mGy*cm (sd. 44.42), mean scan duration 6.86 s (sd. 0.98).

Collimation was 0.6 mm in all scans.

*2023 CT Scan Protocol*

In 2023 using a commercially available container based CT scanner (SOMATOM Scope; Siemens Healthineers) the following non-contrast scan protocols were applied:

For CTH (CT Head): 130 kV, 230 ref. mAs, no exposure modulation applied, pitch 1, mean CTDIvol 53.55 mGy (sd. 0.79), mean DLP 890.27 mGy*cm (sd. 136.92), mean scan duration 25.75 s (sd. 5.99).

In 5 patients an additional fast CTH-scan protocol was used, 2 patients had only the fast CTH scan. To achieve CT scanner speed increase in these 7 patients, in 6 patients collimation was changed from 0.6 to 1.2, in one patient standard CTH pitch was changed from 1.0 to 1.1.

For MFCT (Maxillofacial CT): 110 kV, 35 ref. mAs, exposure modulation applied Z_EC, pitch 0.8, mean CTDIvol 5.50 mGy (sd. 1.04), mean DLP 72.18 mGy*cm (sd. 23.99), mean scan duration 16.87 s (sd. 3.77).

For CSCT (Cervical spine CT): 130 kV, 220 ref. mAs, exposure modulation applied XYZ_EC, pitch 0.65, mean CTDIvol 10.18 mGy (sd. 3.01), mean DLP 177.22 mGy*cm (sd. 59.12), mean scan duration 27.94 s (sd. 4.75).

*2022 and 2023 Image Reconstruction*

Cranial CT: Reconstruction of all images was performed using 512 x 512 image matrix size, axial cranial CT images were reconstructed with 1 mm (2022 increment 0.9 mm, 2023 increment 1mm) and 3 mm (increment 3 mm) slice thickness using brain tissue and bone reconstruction kernels, additional 3 mm coronal and sagittal brain tissue kernel reconstructions were performed (increment 3 mm).

Maxillofacial CT scans were reconstructed with bone kernel in axial, coronal and sagittal plane with 1 mm slice thickness and increment, additional soft tissue kernel reconstructions with slice thickness and increment of 2mm were stored.

Cervical spine: bone and soft tissue kernel reconstructions in axial, sagittal and coronal plane with slice thickness and increment 2 mm were performed, additional axial reconstructions in bone kernel with 1 mm slice thickness were stored, increment for the latter reconstruction was 0.9 mm in the 2022 protocol, 2023 increment was 1 mm.
